# Effect of antiplatelet and anticoagulant medication use on injury severity and mortality in patients with traumatic brain injury treated in the intensive care unit

**DOI:** 10.1007/s00701-023-05850-w

**Published:** 2023-11-01

**Authors:** Juho Vehviläinen, Jyri J. Virta, Markus B. Skrifvars, Matti Reinikainen, Stepani Bendel, Tero Ala-Kokko, Sanna Hoppu, Ruut Laitio, Jari Siironen, Rahul Raj

**Affiliations:** 1 Department of Neurosurgery, Helsinki University Hospital and University of Helsinki, Haartmaninkatu 4, PL320, 00029 HUS Helsinki, Finland; 2 Perioperative and Intensive Care, Division of Intensive Care, Helsinki University Hospital, Helsinki, Finland; 3 Department of Emergency Care and Services, University of Helsinki and Helsinki University Hospital, Helsinki, Finland; 4 Department of Intensive Care, Kuopio University Hospital and University of Eastern Finland, Kuopio, Finland; 5 Department of Intensive Care, Oulu University Hospital and University of Oulu, Oulu, Finland; 6 Department of Intensive Care and Emergency Medicine Services, Tampere University Hospital and Tampere University, Tampere, Finland; 7 Department of Intensive Care, Turku University Hospital and University of Turku, Turku, Finland

**Keywords:** Traumatic brain injury, Intensive care, Antiplatelet medication, Anticoagulant medication, Computed tomography

## Abstract

**Background:**

Antiplatelet and anticoagulant medication are increasingly common and can increase the risks of morbidity and mortality in traumatic brain injury (TBI) patients. Our study aimed to quantify the association of antiplatelet or anticoagulant use in intensive care unit (ICU)–treated TBI patients with 1-year mortality and head CT findings.

**Method:**

We conducted a retrospective, multicenter observational study using the Finnish Intensive Care Consortium database. We included adult TBI patients admitted to four university hospital ICUs during 2003–2013. The patients were followed up until the end of 2016. The national drug reimbursement database provided information on prescribed medication for our study. We used multivariable logistic regression models to assess the association between TBI severity, prescribed antiplatelet and anticoagulant medication, and their association with 1-year mortality.

**Results:**

Of 3031 patients, 128 (4%) had antiplatelet and 342 (11%) anticoagulant medication before their TBI. Clopidogrel (2%) and warfarin (9%) were the most common antiplatelets and anticoagulants. Three patients had direct oral anticoagulant (DOAC) medication. The median age was higher among antiplatelet/anticoagulant users than in non-users (70 years vs. 52 years, *p* < 0.001), and their head CT findings were more severe (median Helsinki CT score 3 vs. 2, *p* < 0.05). In multivariable analysis, antiplatelets (OR 1.62, 95% CI 1.02–2.58) and anticoagulants (OR 1.43, 95% CI 1.06–1.94) were independently associated with higher odds of 1-year mortality. In a sensitivity analysis including only patients over 70, antiplatelets (OR 2.28, 95% CI 1.16–4.22) and anticoagulants (1.50, 95% CI 0.97–2.32) were associated with an increased risk of 1-year mortality.

**Conclusions:**

Both antiplatelet and anticoagulant use before TBI were risk factors in our study for 1-year mortality. Antiplatelet and anticoagulation medication users had a higher radiological intracranial injury burden than non-users defined by the Helsinki CT score. Further investigation on the effect of DOACs on mortality should be done in ICU–treated TBI patients.

**Supplementary Information:**

The online version contains supplementary material available at 10.1007/s00701-023-05850-w.

## Introduction

One of the most common causes of mortality among young people is traumatic brain injury (TBI) [8, 10]. It has been identified as a risk factor for morbidity and mortality among the elderly as well [31]. Coagulopathy, induced by antiplatelet or anticoagulant medication, is often associated in TBI with hematoma progression [19]. As the population ages, the prevalence of these medications has increased [7, 16, 36].

Age and preinjury anticoagulant medication are independent predictors of post-TBI mortality [6, 18]. Antiplatelet medication is associated with a small to non-existent increase in mortality [2, 27]. When compared with TBI severity, there seem to be differences in mortality between different anticoagulants and antiplatelets [6, 18, 22]. Warfarin anticoagulation is associated with a sixfold increase in TBI mortality [6], while direct oral anticoagulants (DOACs) do not seem to increase the risk of in-hospital mortality in mild TBI patients [22, 27]. Furthermore, it seems that the antiplatelet and anticoagulant medications cause no increase in the mortality of trauma patients in the absence of TBI, emphasizing the specific interaction between TBI and antiplatelet and anticoagulant medications [25].

The most prevalent imaging modality in TBI patients is computed tomography (CT). For CT, several prognostic classification and scoring systems have been developed, including the Marshall CT classification [20], the Helsinki CT score [29], the NeuroImaging Radiological Interpretation System (NIRIS) [37], and the Stockholm CT score [23]. The CT scores offer clinicians quantitative and comparable tools to assess TBI severity and estimate the prognosis [34, 35, 39]. Of the developed and validated CT scores, the Helsinki CT has the advantage of being simple while still providing good discrimination and calibration [34, 35].

Both clinical and radiological findings determine the TBI severity. Previous studies have not used CT classification or scoring systems to separate the impact of radiological intracranial injury burden from the effect of antiplatelet or anticoagulant medication. Antiplatelet and anticoagulant medication can cause coagulopathy, thus increasing the TBI burden [19]. Larger lesions should shift the different CT scores to the higher mortality end of the scales, i.e., to higher CT score values [29, 34].

We set out to study whether antiplatelet or anticoagulant medication could affect the severity of TBI in ICU–treated patients and thus impact their 1-year mortality. We hypothesized that (1) the use of antiplatelet medication and anticoagulant medication increases the 1-year mortality of TBI patients, and (2) patients using antiplatelet or anticoagulation medications display a higher radiological intracranial injury burden according to the Helsinki CT score.

## Methods and materials

### Ethical considerations

The ethics committee of Helsinki University Hospital (194/13/03/14 §97), the Finnish National Institute for Health and Welfare (THL/713/5.05.01/2014 and THL/1298/5.05.00/2019), Statistics Finland (TK-53–1047-14), the Social Insurance Institution of Finland (Kela 23/522/2018), the Office of the Data Protection Ombudsman (Dnro 2713/402/2016 28.10.16), and all the participating university hospitals’ research committees approved this study. All committees waived the need for informed patient consent. The study adhered to the Strengthening the Reporting of Observational Studies in Epidemiology (STROBE) guidelines.

### Study design and population

We performed a multicenter retrospective observational study using collected data from the Finnish Intensive Care Consortium (FICC) database. The FICC database is a nationwide database including all ICU–treated patients from the majority of Finnish ICUs [30]. In Finland, all specialized tertiary intensive care of TBI patients is centralized in five university hospital ICUs. Four of these ICUs, covering approximately two-thirds of the population in Finland, participated in the FICC during the study period.

From these four tertiary ICUs, we included all adult TBI patients (age ≥ 18 years) admitted between January 1, 2003, and December 31, 2013 (readmissions excluded). We identified TBI patients by Acute Physiology and Chronic Health Evaluation (APACHE) III diagnostic codes, and the diagnoses were manually verified by screening health records and reviewing primary head computer tomography (CT) scans [28]. We excluded patients if head CT scans or Glasgow Coma Scale (GCS) scores were unavailable. We retrieved data on mortality from the Finnish population register on December 31, 2016 (available for all Finnish residents).

### CT assessment

All patients in the final analysis of our study had a non-contrast CT scan taken at the time of hospital admission. We excluded patients with only post-operative CT scans, CT angiography, or magnetic resonance imaging (MRI) scans. Two authors (J. V. and R. R.) classified the CT images together. No interrater reliability was tested. We chose the Helsinki CT score (Supplementary Table 1) over the other scores due to its simplicity and high performance [34, 35].

### Definition of covariates

We retrieved the medical records of the patients from the FICC database. The GCS score is defined according to the APACHE II definition as the worst measured GCS score during the first ICU day [14]. For intubated and/or sedated patients, the last reliable GCS score preceding sedation is used. The FICC uses a modified version of the World Health Organization/Eastern Cooperative Oncology Group (WHO/ECOG) classification for pre-admission functional status (fit for work or equal, unfit for work but independent in self-care, partially dependent in self-care, totally dependent in self-care) [24]. We defined significant chronic comorbidity according to the APACHE II and Simplified Acute Physiology Score (SAPS) II [14, 15]. Our study defined intracranial pressure (ICP) monitoring through the Therapeutic Intervention Scoring System (TISS) 76, which is routinely collected for the FICC database [13]. We used the NOMESCO classification of Surgical Procedures Finland (NCSP-F) for the definition of an external ventricular drain (EVD, NCSP-F code AAF00), craniotomy for hematoma evacuation (NCSP-F code AAD00, AAD05, AAD15), and for decompressive craniectomy (NCSP-F code AAK80).

### Antiplatelet and anticoagulant medication purchases

In Finland, patients get physician-prescribed medication reimbursed by the Social Insurance Institution with a maximum out-of-pocket payment of roughly 600 euros per calendar year. After reaching the out-of-pocket limit, patients pay 2.50 euros per medication per purchase regardless of the cost.

We obtained data on prescribed and purchased antiplatelet and anticoagulation medication, prior to the TBI, from the Social Insurance Institution Kela from January 1, 2003, to December 31, 2013. Preinjury antiplatelet or anticoagulation user in this study refers to patients who had explicitly been prescribed the medication and who later had purchased the prescribed medication. This method incorporates the assumptions that these patients (1) used the prescribed medication and (2) used the prescribed medication as instructed.

We defined antiplatelet and anticoagulant medication as an Anatomical Therapeutic Chemical (ATC) classification system code of B01AA-C* and B01AE-X* (Supplementary Table 2). We divided patients into three groups: no antiplatelet or anticoagulation medication, antiplatelet medication, and anticoagulation medication.

### Definition of outcome

Our outcome of interest was 1-year all-cause mortality (within 365 days of admission) and additionally mortalities during hospital treatment of the TBI and 30 days from the TBI. Data on death was obtained from Statistics Finland, which upholds a statutory register on all deaths in Finland.

### Statistical analysis

We compared categorical data between groups using a two-sided *χ*^2^ (univariate) test. We present normally distributed data as means with standard deviations (SD) and non-parametric data as medians with interquartile range (IQR). We compared normally distributed data between groups using a *t*-test and non-parametric data using a Mann–Whitney *U* test.

The Helsinki CT score was originally constructed as an ordinal scale; however, due to its several levels and numeric distribution, it can be treated as a continuous variable [29]. To visually demonstrate differences in the Helsinki CT score across groups, we categorized the Helsinki CT score into four groups (− 3 to − 1, 0 to 2, 3 to 7, and 8 to 14, where an increasing score indicates a higher intracranial TBI burden and a higher risk for death).

To assess risk factors for 1-year mortality, we first performed univariate logistic regression analysis yielding odds ratios (OR) with 95% confidence intervals (CI). Then, all statistically significant variables (excluding antiplatelet/anticoagulant use) were included in a multivariable logistic regression model. Finally, antiplatelet/anticoagulant use was added to this model. We report Nagelkerke *R*^2^ for both multivariable models. If antiplatelet and/or anticoagulant medication was significantly associated with mortality in this final model, and if the final model explained more of the variance in the outcome (i.e., if the difference between the log-likelihoods between the models was significant), we considered antiplatelet and/or anticoagulant medication to be independently associated with mortality.

As a sensitivity analysis, we included only patients aged over 70 years to better control for possible differences in age distribution between the groups.

Statistical tools for this study were SPSS IBM Corp., released in 2020. IBM SPSS Statistics for Windows, Version 27.0, Armonk, NY, USA: IBM Corp and STATA StataCorp. 2019. Stata Statistical Software: Release 17. College Station, TX: StataCorp LLC.

## Results

### Patient characteristics

**Fig. 1:**
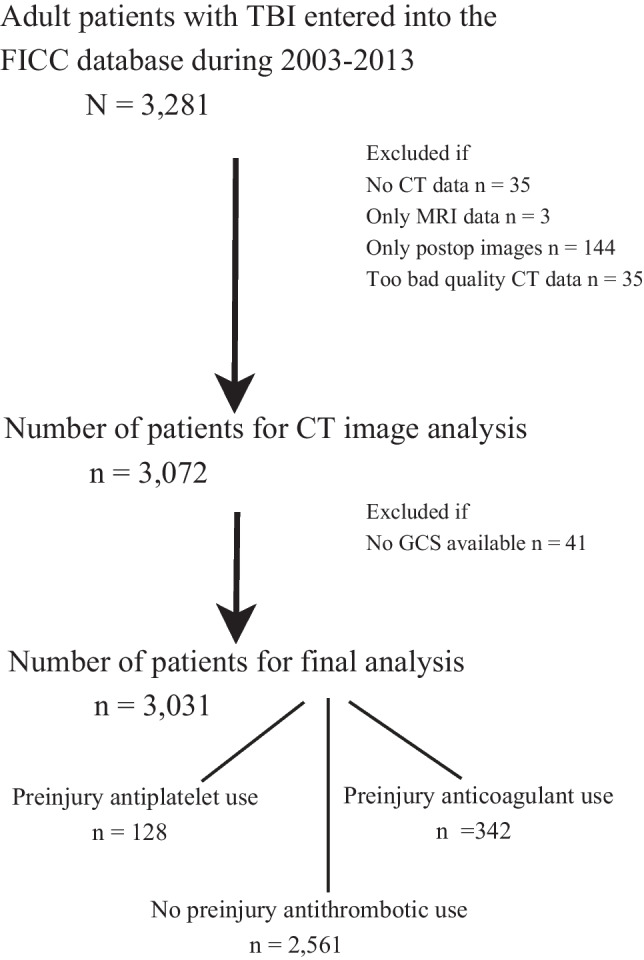
Study patient flow chart. Abbreviations: *TBI* traumatic brain injury; *FICC* Finish Intensive Care Consortium; *CT* computed tomography; *GCS* Glasgow Coma Scale

Of 3031 patients in our study, 128 (4%) had preinjury antiplatelet medication, 342 (11%) had preinjury anticoagulation medication (Fig. 1), and 2561 (85%) did not have antiplatelet or anticoagulation medication. None of the patients had both antiplatelet and anticoagulation medication. Types of medications are listed in Supplementary Table 2. The most common anticoagulant medication was warfarin (*n* = 270, 9%), and the most common antiplatelet medication was clopidogrel (*n* = 60, 2%). Three patients used DOACs.

**Fig. 2:**
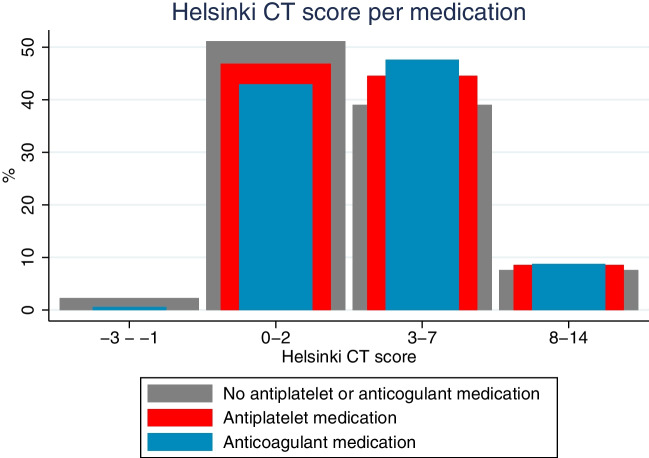
Percentages of Helsinki CT score subgroups (− 3 to − 1; 0–2; 3–7; 8–14) divided by preinjury antiplatelet and anticoagulant medication use. Note that the change in bar width is only for presentation purposes. The patients without preinjury antiplatelet or anticoagulant medication had statistically significant differences from patients using anticoagulant medication in the Helsinki CT score subgroups 0–2 (*p* = 0.014) and 3–7 (*p* = 0.006)

Patients with antiplatelet or anticoagulant medication had higher median age (no medication 52 years vs. antiplatelet/anticoagulation 70 years), were less frequently fit for work (no medication 65% vs. antiplatelet 31% vs. anticoagulation 33%), had more frequently significant chronic comorbidity (no medication 7% vs. antiplatelet 20% vs. anticoagulation 14%), higher SAPS II scores (Table 1), and more severe admission head CT findings (Fig. 2). The median of the Helsinki CT score was higher in the antiplatelet and anticoagulation medication groups than in the no-medication group (Fig. 2, Table 1).

**Table 1:** Baseline characteristics and treatment of traumatic brain injury patients

Variables	All patients (*N* = 3031)	No antiplatelet or anticoagulation medication (*n* = 2561)	Preinjury antiplatelet medication (*n* = 128)	*p*-value#	Preinjury anticoagulant medication (*n* = 342)	*p*-value¶
Age, median (IQR)	55 (41, 67)	52 (37, 63)	70 (60, 78)	< 0.001	70 (61, 76)	< 0.001
18–40 years	753 (25%)	743 (29%)	0 (0%)	< 0.001	10 (3%)	< 0.001
41–64 years	1319 (47%)	1263 (49%)	46 (36%)		110 (32%)	
≥ 65 years	859 (28%)	555 (22%)	82 (64%)		222 (65%)	
GCS score, median (IQR)	9 (5, 14)	9 (5, 14)	10 (5, 14)	0.130	8 (5, 13)	0.065
3–8	1410 (47%)	1189 (46%)	47 (37%)	0.036	174 (51%)	0.022
9–12	585 (19%)	477 (19%)	34 (26%)		74 (22%)	
13–15	1036 (34%)	895 (35%)	47 (37%)		94 (27%)	
Women	676 (22%)	543 (21%)	27 (21%)	0.977	106 (31%)	< 0.001
Pre-admission performance status*, **						
Fit for work or equal	1827 (60%)	1674 (65%)	40 (31%)	< 0.001	113 (33%)	< 0.001
Unfit for work, but independent in self-care	908 (30%)	673 (26%)	67 (52%)		168 (49%)	
Partially dependent in self-care	174 (6%)	108 (4%)	16 (13%)		50 (15%)	
Totally dependent in self-care	48 (2%)	32 (1%)	5 (4%)		11 (3%)	
Significant chronic comorbidity^†^	252 (8%)	178 (7%)	25 (20%)	< 0.001	49 (14%)	< 0.001
SAPS II score, median (IQR)	34 (23, 50)	33 (22, 48)	38 (26, 55)	< 0.001	43 (30, 58)	< 0.001
Modified SAPS II^‡^, median (IQR)	16 (11, 21)	16 (11, 21)	16 (10, 22)	0.367	17 (13, 17)	0.006
Helsinki CT score, median (IQR)	2 (2, 4)	2 (2, 4)	3 (2, 4)	0.043	3 (2, 5)	< 0.001
Craniotomy and hematoma evacuation	1160 (38%)	918 (36%)	56 (44%)	0.069	186 (54%)	< 0.001
Decompressive craniectomy	49 (2%)	45 (2%)	1 (1%)	0.406	3 (1%)	0.231
External ventricular drain	158 (5%)	143 (6%)	4 (3%)	0.232	11 (3%)	0.067
ICP monitoring	711 (23%)	627 (24%)	19 (15%)	0.013	65 (19%)	0.026
Mechanical ventilation	1990 (66%)	1657 (65%)	81 (63%)	0.743	252 (74%)	0.001
LOS ICU, days, median (IQR)	2 (1, 4)	2 (1, 4)	1 (1, 3)	0.083	2 (1, 4)	0.947
LOS hospital, days, median (IQR)	6 (3, 11)	6 (3, 11)	5 (3, 9)	0.018	5 (3, 9)	0.011
Hospital mortality	389 (13%)	305 (12%)	16 (13%)	0.843	68 (20%)	< 0.001
30–day mortality	581 (19%)	436 (17%)	34 (27%)	0.006	111 (32%)	< 0.001
1-year mortality	770 (25%)	574 (22%)	52 (41%)	< 0.001	144 (42%)	< 0.001

Abbreviations: *APACHE* Acute Physiology, and Chronic Health Evaluation; *GCS* Glasgow Coma Scale; *LOS* length of stay; *ICP* intracranial pressure; *ICU* intensive care unit; *SAPS* Simplified Acute Physiology Score; *TBI* traumatic brain injury

^**#**^No antiplatelet or anticoagulation vs. antiplatelet medication

^**¶**^No antiplatelet or anticoagulation vs. anticoagulation medication

^*^A modified World Health Organization/Eastern Cooperative Oncology Group classification system implemented by the Finnish Intensive Care Consortium

^**^Not available for 74 patients

^†^Any chronic comorbidity according to APACHE II or SAPS II

^‡^SAPS II score without age, GCS, and chronic diseases

### Differences in mortality

The unadjusted 1-year mortality rate was higher in both antiplatelet and anticoagulant medication study populations (no medication 22% vs. antiplatelet 41% vs. anticoagulation 42%) (Table 1). The results of the univariate logistic regression analyses are shown in Table 2. Crude hospital mortality was higher in the preinjury anticoagulative medication group, whereas crude 30-day mortality was higher in both the preinjury antiplatelet medication and anticoagulant medication groups than in the no medication group (Table 1).

**Table 2:** Univariate and multivariable logistic regression models showing an association between patient demographics, markers of traumatic brain injury severity, and 1-year mortality

Variable	Univariate logistic regression		Multivariable logistic regression without antiplatelet or anticoagulant medication		Multivariable logistic regression with antiplatelet and anticoagulant medication	
	Odds ratio (95% CI)	*p*-value	Odds ratio (95% CI)	*p*-value	Odds ratio (95% CI)	*p*-value
Age	1.04 (1.04–1.05)	< 0.001	1.04 (1.04–1.05)	< 0.001	1.04 (1.03–1.05)	< 0.001
Sex						
Male	1.0		1.0		1.0	
Female	1.23 (1.02–1.49)	0.033	0.90 (0.70–1.15)	0.396	0.90 (0.70–1.16)	0.416
GCS score	0.77 (0.75–0.79)	< 0.001	0.82 (0.80–0.85)	< 0.001	0.82 (0.80–0.85)	< 0.001
Significant comorbidity	2.72 (2.09–3.54)	< 0.001	1.84 (1.31–2.58)	< 0.001	1.79 (1.28–2.52)	< 0.001
Pre-admission functional status						
Independent in ADL	1.0		1.0		1.0	
Dependent in ADL	3.42 (2.59–4.51)	< 0.001	1.85 (1.31–2.60)	< 0.001	1.78 (1.26–2.51)	0.001
Modified SAPS II score*	1.13 (1.11–1.14)	< 0.001	1.08 (1.06–1.09)	< 0.001	1.08 (1.06–1.09)	< 0.001
Helsinki CT score	1.39 (1.34–1.44)	< 0.001	1.19 (1.14–1.24)	< 0.001	1.19 (1.14–1.24)	< 0.001
Pre-TBI antiplatelet medication	2.37 (1.64–3.41)	< 0.001	-	-	1.62 (1.02–2.58)	0.043
Pre-TBI anticoagulant medication	2.52 (1.99–3.18)	< 0.001	-	-	1.43 (1.06–1.94)	0.019

Univariate model variables with *p*-value < 0.05 were included in the multivariable model

Abbreviations: *ADL* activities of daily living; *CI* confidence interval; *GCS* Glasgow Coma Scale; *SAPS* Simplified Acute Physiology Score; *TBI* traumatic brain injury

^*^SAPS II score without age, GCS, and chronic diseases

In the multivariable model including both antiplatelet and anticoagulation medication, higher odds for 1-year mortality were associated with higher age, significant comorbidity, higher Helsinki CT score, preinjury antiplatelet medication, and preinjury anticoagulant medication. A higher GCS score was associated with lower odds for 1-year mortality (Table 2). The multivariable model without antiplatelet or anticoagulative medication had the same associations excluding the medications (Table 2). The multivariable models with the endpoints of hospital mortality (Supplementary Table 3) and 30-day mortality (Supplementary Table 4) had an association only with preinjury anticoagulation medication.

The full multivariable model with both medications had a Nagelkerke *R*^2^ of 0.322, and the model without antiplatelet or anticoagulant medication had a Nagelkerke *R*^2^ of 0.320. The two models differed slightly in their performance: *χ*^2^ test 8.16, *p* = 0.0169, indicating better goodness-of-fit for the full model with antiplatelet and anticoagulant medication.

The sensitivity analysis included only patients who were older than 70 years (*n* = 564). The analysis indicated increased odds for 1-year mortality for age (OR 1.09, 95% CI 1.05–1.14), preinjury antiplatelet medication (OR 2.28, 95% CI 1.20–4.33), and preinjury anticoagulant medication (OR 1.50, 95% CI 0.97–2.32) when compared to the odds of 1-year mortality across all age groups (Supplementary Table 5).

## Discussion

### Key findings

In this large multicenter observational study including 3031 ICU–treated TBI patients, prescribed preinjury antiplatelet and preinjury anticoagulation medication was associated with an increased risk for 1-year mortality. The prevalence of antiplatelet and anticoagulation medication use increases with age [7, 16, 36], and age is also a risk factor for more severe TBIs [6, 18]. Even after adjusting for age, GCS, and radiological findings, preinjury use of antiplatelet or anticoagulation medication was associated with an increased risk of death both in the initial and in the sensitivity analysis including only patients over 70. The increased risk of death could be linked to fragility or, in theory, be due to coagulopathy and the progression of intracranial hemorrhage. Our study classifies the preinjury medication users as those patients who had been prescribed antiplatelet or anticoagulant medication. This limitation places the patients using over-the-counter acetylsalicylic acid into the control group. Presumably, the indications for prescribed antiplatelet and over-the-counter acetylsalicylic acid are different.

One-year mortality in antiplatelet and anticoagulant medication groups was similar (41% and 42%), as was the increase in the risk of death in multivariable models. Our sensitivity analysis, including only patients over 70, showed similar results. Short-term mortality (during the index hospitalization and within 30 days) after TBI was higher only in the anticoagulant medication study population. Significant comorbidity or pre-admission functional status was not associated with the short-time mortalities, but the Helsinki CT score was. This suggests that in the preinjury anticoagulation medication study population, the injury extent and the hematoma size were more dominating factors than in the preinjury antiplatelet medication population when considering the short-time mortality.

The antiplatelet medication group had a higher chronic comorbidity prevalence versus the anticoagulation group (20% vs. 14%). Our initial assumption was that anticoagulation medication should increase 1-year mortality more than antiplatelet medication; however, our data give no support to this. Higher chronic comorbidity prevalence and a high proportion of secondary cardiovascular prevention (clopidogrel as the most common medication) in the antiplatelet medication group might influence this.

The Helsinki CT score served as our proxy for radiological intracranial injury burden. Anticoagulant and antiplatelet medication use was associated with higher Helsinki CT scores, indicating a more severe radiological intracranial injury burden. However, as anticoagulants and antiplatelets were associated with increased 1-year mortality even in a model controlling for initial CT findings, the increased mortality risk is not explained solely by a more severe initial radiological intracranial injury burden.

The effect of the antiplatelet or the anticoagulation medication can cause coagulopathy, resulting in intracranial hemorrhage which is often fatal [26]. The antithrombotic effect of the antiplatelets can in part be reversed by the use of platelet transfusion. For warfarin, the counteragents are prothrombin complex concentrates (PCC) and vitamin K. The anticoagulative effects of DOACs are more difficult to reverse, but certain high-priced specific counteragents have been developed [1, 17]. DOACs, however, lower the risk of spontaneous intracranial hemorrhages when compared to warfarin [32].

### Comparison to previous studies

Our results align with earlier studies looking at the association between preinjury anticoagulation medication and mortality [6, 18, 27]. Other studies have found differences in radiological intracranial injury burden (extra-axial lesion progression) with patients using preinjury antiplatelet or anticoagulant medication, but no effect in mortality [21]. The number of patients using DOACs in this study was too small to draw any conclusions, but there are indications that preinjury use of DOACs has a lower risk of intracranial hemorrhage than warfarin [3, 27].

Preinjury antiplatelet medication’s effect on mortality ranges in the literature from significant to small to non-existent [2, 11, 27, 33]. Our results indicate that preinjury antiplatelet medication increases the risk of 1-year mortality. This is especially profound in the sensitivity analysis including only patients who were older than 70, although this may partly be explained by the high prevalence of chronic comorbidities among antiplatelet users.

The prevalence of antiplatelet (4%) and anticoagulant (11%) medication was similar in our study than in other studies. A Finnish study, including mild TBIs, reported a slightly smaller prevalence of preinjury anticoagulant medication (8%) [27]. A study from the USA [6] found a similar (11%) prevalence of anticoagulant medication, whereas a recent study [38] from China reported that approximately 12% of older TBI patients had preinjury anticoagulant medication and 13% antiplatelet medication.

An increased need for neurosurgical procedures [11, 12] has been reported by some studies while others have not found any effect [4, 5, 21, 33] with preinjury antiplatelet or anticoagulant medication. In our study, craniotomy and hematoma evacuations were more common in patients who had used anticoagulant medication, whereas the use of ICP monitoring was lower in both antiplatelet and anticoagulation groups (Table 1). This could indicate more extra-axial lesions (seen in [21]) that benefit from evacuation than the more diffuse injuries.

### Strengths and limitations

We used a large multicenter high-quality database to collect data prospectively. Thus, we were able to include more than three thousand patients in our study. There was a small amount of missing data, and we had a complete 12-month mortality follow-up. Our patient cohort also represents well the general ICU–treated TBI population in Finland as the referral population of the four neuro-ICUs is approximately 3.5 million people, encompassing two-thirds of the Finnish population. To our knowledge, no previous studies link the antiplatelet and anticoagulant medication with prognostic head CT scoring systems.

We acknowledge some limitations in this study. The FICC is a general ICU database and lacks some TBI-specific parameters, like admission GCS score, pupillary light reactivity, and specific neurosurgical procedures. Data on pupillary light responses would probably have improved the predictive performance of our multivariable model [34]. Furthermore, we were not able to account for antiplatelet/anticoagulation medication dosage, indications, the level of inhibition, reversal strategy, or its timing. Our data is from 2003 to 2013, and the use of DOACs has been steadily on the rise [9]. In this regard, our analysis should be done using more recent data. Unfortunately, such an extensive data set with different CT classifications was not available. We highlight that our study included patients treated in tertiary hospital ICUs and did not include patients not referred to such ICUs or patients with milder TBIs. Neurological outcome was not available in our dataset; thus, 1-year mortality was the endpoint for this study.

An inherent limitation of a retrospective study is that only associations can be found, and it is not possible to prove whether the relationship between the predictive factor and outcome is of causal nature. It is plausible that medications interfering with hemostasis may cause more severe trauma-related bleeding and thus worsen the outcomes of TBI patients. However, it is also possible that our results may be explained by selection bias: patients taking anticoagulant or antiplatelet medications may have poorer underlying health than patients not taking these medications, which may be the true reason for the difference in outcome.

Our data is limited by the distribution of different antiplatelet medications. Acetylsalicylic acid—used for primary and secondary cardiovascular prevention—is available as an over-the-counter medication in Finland and thus is absent from our data drawn from the Social Insurance Institution Kela database. In contrast, other antiplatelets (i.e., clopidogrel, dipyridamole, and prasugrel) are mostly for secondary prevention after ischemic stroke or following percutaneous coronary intervention. We acknowledge that the possible missing acetylsalicylic acid information on some patients is a major limitation for the antiplatelet medication part of this study. It is possible that patients in the antiplatelet group had more severe co-morbidities, and possibly we were not able to sufficiently control for this in our statistical analyses.

We did not account for out-of-hospital TBI–related deaths. It is possible that a larger number of antiplatelet and anticoagulation users died before reaching the hospital due to more severe intracranial bleeds.

## Conclusion

The use of prescribed preinjury antiplatelet or anticoagulant medication is an independent risk factor for 1-year mortality in ICU–treated TBI patients. These medications are on the rise due to increasing life expectancy [7, 16, 36]. TBI patients with antiplatelet or anticoagulant medication had higher Helsinki CT scores reflecting a higher radiological intracranial injury burden, but this alone did not explain the increased mortality in patients taking antiplatelet or anticoagulant medication before the injury. Further studies are needed to assess the effects of DOAC on TBI patients.

### Supplementary Information

Below is the link to the electronic supplementary material.

Supplementary file1 (DOCX 16 KB)

Supplementary file2 (DOCX 17 KB)

Supplementary file3 (DOCX 19 KB)

Supplementary file4 (DOCX 20 KB)

Supplementary file5 (DOCX 19 KB)

Supplementary file6 (DOCX 10 KB)

## Data Availability

The datasets analyzed during the current study are not publicly available due to restrictions based on the General Data Protection Regulation (GDPR) on sensitive data such as personal health data. Access to the data may be requested through the Finnish Institute for Health and Welfare (THL) Biobank (https://thl.fi/en/web/thl-biobank/for-researchers).

## Code availability

Relevant Stata codes used for the statistical analysis are within the Supplementary material (stata_codes.do).
