# Supplementary material for: Effect of antiplatelet and anticoagulant medication use on injury severity and mortality in patients with traumatic brain injury treated in the intensive care unit
Source: Acta Neurochir (Wien). 2023 Nov 1;165(12):4003–12. doi: 10.1007/s00701-023-05850-w (PMC10739466; doi:10.1007/s00701-023-05850-w)
Supplement: Supplementary file 2 — Supplementary file2 (DOCX 17 KB) [file 701_2023_5850_MOESM2_ESM.docx]

**Effect of antiplatelet and anticoagulant medication use on injury severity and mortality in patients with traumatic brain injury treated in the intensive care unit**

Juho Vehviläinen*, MD, MSc ^1^, Jyri J. Virta, MD, PhD ^2^, Markus B. Skrifvars, MD, PhD ^3^, Matti Reinikainen, MD, PhD ^4^, Stepani Bendel, MD, PhD ^4^, Tero Ala-Kokko, MD, PhD ^5^, Sanna Hoppu, MD, PhD ^6^, Ruut Laitio, MD, PhD ^7^, Jari Siironen, MD, PhD, ^1^ Rahul Raj, MD, PhD ^1^

1. Department of Neurosurgery, Helsinki University Hospital and University of Helsinki, Helsinki, Finland
2. Perioperative and Intensive Care, Division of Intensive Care, Helsinki University Hospital, Finland
3. Department of Emergency Care and Services, University of Helsinki and Helsinki University Hospital, Helsinki, Finland.
4. Department of Intensive Care, Kuopio University Hospital & University of Eastern Finland, Kuopio, Finland
5. Department of Intensive Care, Oulu University Hospital & University of Oulu, Oulu, Finland
6. Department of Intensive Care and Emergency Medicine Services, Tampere University Hospital & University of Tampere, Tampere, Finland
7. Department of Intensive Care, Turku University Hospital & University of Turku, Turku, Finland

Corresponding author*: **Juho Vehviläinen**

- E-mail: juho.vehvilainen@helsinki.fi

| **Supplementary Table 2:** Antiplatelets and anticoagulants used by the study population pre-TBI | |
| --- | --- |
| **Drug** (generic name and ATC code) | **Number of patients using the drug** (% of antiplatelets or anticoagulants, % of all patients in the study N = 3,031) |
| **Antiplatelets** | 128 (27%^*^, 4%) |
| clopidrogrel (B01AC04) | 60 (47%^#^, 2%) |
| dipyridamole (B01AC07) | 22 (17%^#^, 0.7%) |
| prasugrel (B01AC22) | 1 (1%^#^, 0.03%) |
| acetylsalicylic acid (B01AC30) | 45 (35%^#^, 1%) |
| **Anticoagulants** | 342 (73%^*^, 11%) |
| warfarin (B01AA03) | 270 (79%**^¶^**, 9%) |
| dalteparin (B01AB04) | 20 (6%**^¶^**, 0.7%) |
| enoxaparin (B01AB05) | 45 (13%**^¶^**, 1%) |
| dabigatran etexilate (B01AE07) | 2 (0.6%**^¶^**, 0.07%) |
| rivarozaban (B01AF01) | 1 (0.3%**^¶^**, 0.03%) |
| fondaparinux (B01AX05) | 4 (1.2%**^¶^**, 0.1%) |
| ^*^from the total number of antiplatelet and anticoagulant drugs n = 470  ^#^ from the total number of antiplatelet drugs n = 128  **^¶^**from the total number of anticoagulant drugs n = 342 | |
