# Supplementary material for: Effect of antiplatelet and anticoagulant medication use on injury severity and mortality in patients with traumatic brain injury treated in the intensive care unit
Source: Acta Neurochir (Wien). 2023 Nov 1;165(12):4003–12. doi: 10.1007/s00701-023-05850-w (PMC10739466; doi:10.1007/s00701-023-05850-w)
Supplement: Supplementary file 5 — Supplementary file5 (DOCX 19 KB) [file 701_2023_5850_MOESM5_ESM.docx]

**Effect of antiplatelet and anticoagulant medication use on injury severity and mortality in patients with traumatic brain injury treated in the intensive care unit**

Juho Vehviläinen*, MD, MSc ^1^, Jyri J. Virta, MD, PhD ^2^, Markus B. Skrifvars, MD, PhD ^3^, Matti Reinikainen, MD, PhD ^4^, Stepani Bendel, MD, PhD ^4^, Tero Ala-Kokko, MD, PhD ^5^, Sanna Hoppu, MD, PhD ^6^, Ruut Laitio, MD, PhD ^7^, Jari Siironen, MD, PhD, ^1^ Rahul Raj, MD, PhD ^1^

1. Department of Neurosurgery, Helsinki University Hospital and University of Helsinki, Helsinki, Finland
2. Perioperative and Intensive Care, Division of Intensive Care, Helsinki University Hospital, Finland
3. Department of Emergency Care and Services, University of Helsinki and Helsinki University Hospital, Helsinki, Finland.
4. Department of Intensive Care, Kuopio University Hospital & University of Eastern Finland, Kuopio, Finland
5. Department of Intensive Care, Oulu University Hospital & University of Oulu, Oulu, Finland
6. Department of Intensive Care and Emergency Medicine Services, Tampere University Hospital & University of Tampere, Tampere, Finland
7. Department of Intensive Care, Turku University Hospital & University of Turku, Turku, Finland

Corresponding author*: **Juho Vehviläinen**

- E-mail: juho.vehvilainen@helsinki.fi

| **Supplementary Table 5:** Univariate and multivariable logistic regression models showing the association between patient demographics, traumatic brain injury severity markers, and one-year mortality in patients over 70 years. | | | | | | |
| --- | --- | --- | --- | --- | --- | --- |
| **Variable** | **Univariate logistic regression** | | **Multivariable logistic regression without antiplatelet or anticoagulant medication** | | **Multivariable logistic regression with antiplatelet and anticoagulant medication** | |
|  | **Odds ratio (95% CI)** | **p-Value** | **Odds ratio (95% CI)** | **p-Value** | **Odds ratio (95% CI)** | **p-Value** |
| **Age** | 1.08 (1.04-1.12) | <0.001 | 1.10 (1.05-1.14) | <0.001 | 1.09 (1.05-1.14) | <0.001 |
| **Sex** |  |  |  |  |  |  |
| Male | 1.0 |  |  |  |  |  |
| Female | 1.03 (0.73-1.46) | 0.857 |  |  |  |  |
| **GCS score** | 0.79 (0.75-0.82) | <0.001 | 0.82 (0.77-0.86) | <0.001 | 0.81 (0.77-0.86) | <0.001 |
| **Significant comorbidity** | 2.05 (1.29-3.26) | 0.002 | 1.94 (1.12-3.34) | 0.018 | 1.84 (1.04-3.19) | 0.030 |
| **Pre-admission functional status** |  |  |  |  |  |  |
| Independent in ADL | 1.0 |  | 1.0 |  | 1.0 |  |
| Dependent in ADL | 1.96 (1.29-2.97) | 0.002 | 1.50 (0.93-2.41) | 0.095 | 1.49 (0.92-2.40) | 0.104 |
| **Modified SAPS II score** | 1.09 (1.07-1.12) | <0.001 | 1.05 (1.02-1.08) | 0.002 | 1.05 (1.01-1.08) | 0.002 |
| **Helsinki CT score** | 1.18 (1.10-1.27) | <0.001 | 1.09 (1.00-1.19) | 0.045 | 1.10 (1.01-1.20) | 0.026 |
| **Pre-TBI antiplatelet medication** | 1.88 (1.09-3.24) | 0.022 | - | - | 2.28 (1.20-4.33) | 0.012 |
| **Pre-TBI anticoagulant medication** | 1.61 (1.11-2.33) | 0.013 | - | - | 1.50 (0.97-2.32) | 0.067 |
| ***** SAPS II score without age, GCS, and chronic diseases.  Univariate model variables with p-Value < 0.05 were included in the multivariable model.  *Abbreviations*: ADL, Activities of Daily Living; CI, Confidence Interval; GCS, Glasgow Coma Scale; SAPS, Simplified Acute Physiology Score; TBI, Traumatic Brain Injury | | | | | | |
